# Contraceptive vaginal ring experiences among women and men in Kisumu, Kenya: A qualitative study

**DOI:** 10.15761/FWH.1000122

**Published:** 2017-02-16

**Authors:** E McLellan-Lemal, K Ondeng’e, DA Gust, M Desai, FO Otieno, PA Madiega, B Nyagol, EM Makanga

**Affiliations:** 1 Centers for Disease Control and Prevention, Office of Infectious Diseases, National Center for HIV/AIDS, Viral Hepatitis, STD and TB Prevention, Division of HIV/AIDS Prevention, Atlanta, Georgia-USA; 2 Kenya Medical Research Institute, Kisumu, Kenya; 3 Nyanza Reproductive Health Society, Kisumu, Keny

**Keywords:** contraceptive ring, Kenya, women, men, qualitative, lived experience

## Abstract

**Background:**

Future HIV prevention options for women will likely include Antiretroviral (ARV)-based intravaginal rings. Valuable insights may be gained by examining user experiences with a similar licensed technology, a contraceptive ring, especially in settings where this technology may not be currently available.

**Methods:**

In-depth interviews with 24 females enrolled in a trial assessing acceptability and use of a contraceptive ring, and 20 male sexual partners were conducted September 2014–April 2015. Elements of ethnography and phenomenological anthropology were used to collect, analyze, interpret, and describe ring users’ experiences. Thematic analysis was completed in MaxQDA-10.

**Results:**

Experiences with the contraceptive ring reflected a broader Family Planning (FP) paradigm that centered around three themes: latitudes and drawbacks of FP (being free); an FP method needs to be compatible with a woman’s body (feeling normal); and dealing with fertility control uncertainties (how well does it really work). FP intentions and disclosure practices were influenced by partner support, socioeconomic factors, religion, cultural beliefs, and societal norms, including female sexuality. A user-friendly FP design was emphasized. Non-suppression of menstruation was favored by most. Unease with vaginal insertion as well as ring placement issues (slippage, expulsion) created initial challenges requiring clinician assistance and practice for some participants. While minor side-effects were described, concerns centered on ring efficacy, negative effect on a woman’s sexual desire, and future fertility issues.

**Conclusions:**

Awareness of the multiple contexts in ring users’ experience may inform the development, education, and promotion approaches for future ARV rings.

## Introduction

Heterosexual sex is the primary mode of HIV acquisition among women regardless of age. As a consequence of sexual intercourse without a condom, women may also be at increased risk of unintended pregnancies as well as sexually transmitted infections [1]. Women represent an estimated 52% of HIV cases in low- and middle-income countries worldwide [2]. Disproportionately, women 15–24 years of age account for 60% of new HIV cases among young people globally [3]. An estimated 80% of these young women reside in sub-Saharan Africa [3].

Intravaginal rings (henceforth referred to as rings) can deliver hormones safely and effectively for contraception and management of menopausal symptoms. Antiretroviral-based (ARV) rings hold promise for increasing HIV prevention options for women. In sub-Saharan Africa, the availability of rings is either limited or non-existent [4]. Given the importance of acceptability and adherence in determining the efficacy of ARV-based rings with and without contraception co-formulation in clinical trials and in demonstrating effectiveness following licensure and implementation, a better understanding of potential ring use facilitators and barriers are needed [5].

Acceptability studies and clinical trials conducted in selected countries in sub-Saharan Africa have suggested high acceptance of rings. Acceptability, however, may not be a sufficient indicator of actual use. Both contraceptive and microbicide research have suggested that relationship and situational contextual factors, rather than acceptability, may provide better insights regarding product use and patterns of use [6,7]. In addition, the idea that product acceptability and adherence in clinic trials should be examined as separate constructs has been proposed [8]. Montgomery and colleagues [7] astutely point out that biomedical framing of acceptability in terms of product properties, willingness to use, and risk reduction is limiting, namely because technology, be it contraceptive- or HIV microbicide-oriented, fails to recognize the “more holistic picture of women and men’s sexuality and sexual health” (p. 649). Others have also proposed that research must explore contraceptives ecologically within sexual, social, and cultural contexts [9]. Emphasis should optimally be on discerning factors that may influence sexual acceptability of contraceptives at macro (gender, cultural, race/ethnicity, place, inequality, and structure), social (relationship and partner factors), and individual levels [9].

Qualitative research examining the structure of the “lived experience” may help increase researcher and public health practitioner understandings as to how rings fit with and can potentially influence sexual and reproductive health knowledge, attitudes, and behaviors. A “live experience” approach may also help to inform future ARV ring properties, educational messages, adherence assessment and counseling tools, plus partner engagement strategies. As noted by Gammeltoft [10] “to improve women’s health and quality of life, it seems important to enhance our insights into the ways in which women themselves perceive and use fertility control technologies and to better understand how the uses of technology mediate social relationships and cultural meanings” (p.3). Hence, we undertook in-depth interviews with women and their sexual partners in Kisumu, Kenya to explore ring users’ experiences, and the meaning that participants themselves made of their ring use experiences, within the context of their everyday lives, as well as within the temporality of their research participation.

### Ethics statement

The Kenya Medical Research Institute Scientific and Ethical Review Unit and an Institutional Review Board for the United States Centers for Disease Control and Prevention, reviewed and approved the study protocol, informed consent, and interview guide. A request for waiver of documentation of informed consent was approved, and a verbal informed consent process was used. Consent was attained in the language preferred by the participant (English, Kiswahili, or Dholuo) prior to data collection. Participants received a bar of soap as honorarium for completing an in-depth interview plus 500 Kenya Shillings (approximately $5 USD) for transport. All participants received light refreshments consisting of a soft drink and tea biscuits during the data collection.

## Methods

We incorporated elements of ethnography and phenomenological anthropology to collect, analyze, interpret, and describe ring users’ experiences and perspectives. While ethnography focuses on discovering shared cultural meanings, phenomenology in anthropology demonstrates how experiences of and perceptions about everyday things and events are constructed through social and practical actions [11,12]. Both approaches emphasize the insider (emic) perspective and take into account that the lived experience is multidimensional in that a number of different aspects (e.g., sensorial, corporeal, political, ethical, relational) occur simultaneously and influence the meaning derived from and assigned to an experience. Methodologically, this means that our intent was to achieve an understanding of experiential phenomena on their own terms, which could potentially be different from what we might focus on in our clinical and behavioral assessment research tools be they contraceptive- or microbicide-oriented.

**Figure 1:**
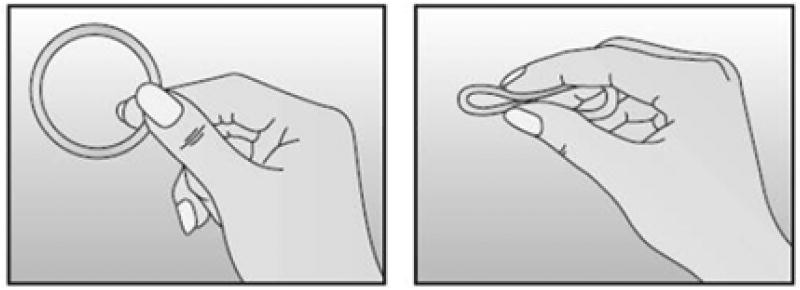
Image of the contraceptive vaginal ring, NuvaRing®.

In-depth interviews were conduct between September 2014 and April 2015 as part of a qualitative sub study with in a single-group observational study examining the acceptability, adherence and biologic effects of NuvaRing^®^ (see Figure 1) in Kisumu, Kenya [13]. We purposively selected 20 women for a one-time in-depth interview if they had completed at least three consecutive months of ring use and had a male sexual partner from the last 30 days who was also willing to take part in an individual interview. Male sexual partners had to be at least 18 years of age, fluent in English, Swahili, or Dholuo, and willing to take part in an audio-recorded interview. In addition, we interviewed four out of five women who became pregnant during the ring-use phase of the main study (regardless of the actual length of time on the ring before their pregnancy diagnosis and partner willingness to complete an interview). None were willing to ask their partners to participate in an interview. The fifth woman was lost to follow-up.

Interviewers who were members of the local community and fluent in all three data collection languages conducted the interviews. The interviewers completed two-week training on in-depth interviewing techniques, as well as refresher training, before conducting interviews. Female interviewers conducted all interviews with female participants. Where possible, male interviewers interviewed male sexual partners. Each one-on-one interview lasted about one hour.

A semi-structured interview guide containing descriptive questions [14] was translated from English into Swahili and Dholuo and then back-translated into English. The interview guide included questions on general knowledge, beliefs, and experiences regarding contraception; influences on contraceptive method choice, contraception decision-making communication and roles; and views on and experiences with using a contraceptive ring. We administered a brief demographic questionnaire to male participants and extracted comparable demographic information from the main study data for female participants.

All interviews were audio-recorded, transcribed verbatim in their original data collection language, and reviewed for accuracy using a transcription protocol [15]. Non-English transcripts then underwent a meaning-based translation and verification process. A thematic data analysis following phenomenological procedures described by Hycner [16] was completed on the English-based transcripts in MaxQDA-10 (VERBI Software – Consult – Sozialforschung GmbH, Berlin, Germany).

Our findings include quotes to help elucidate particular experiences and perspectives, albeit the meaning-based English translations may not adequately capture linguistic nuances/richness contained in the original interview language. To minimize the potential for inadvertent identification of study participants, we provide minimal participant information. Moreover, to avoid too many quotes from a single participant or greater weight/representation given to a particular interview, we limited each interview to a maximum of two quote extractions. Not all interviews, however, produced quotes.

## Results

The mean age for the 24 women interviewed was 25 (range 18–34), while the mean age for the 20 men was 33 (range: 18–58). As shown in Table 1, 79.5% of all participants were Luo and 86.4% were married or living as married. Men were more likely to have completed secondary education or higher than were women, 75.0% vs 35.7%, respectively.

The terms contraception and birth control were not part of interview participants’ vocabulary. All participants referred to methods for delaying or avoiding pregnancy as family planning regardless if their expressed desires were to stop having children altogether, to limit the number of children they had (controlling the family size), or to space births by several years. The women described a range of modern family planning approaches available to them and openly discussed switching from one method to another, mostly due to unpleasant side effects. While a few men were knowledgeable about family planning methods, most had limited information, or were predominately concerned with potential side effects including loss of sexual interest by their partners.

### Being free: Latitudes and drawbacks of family planning

Participants acknowledged that women and men often held different and sometimes conflicting views on family planning. Some participants regarded family planning as an individual choice that varied across community members and possibly their circumstances (e.g., economic status, marital situation, number of children already in the household). Most expressed that communication around family planning and other women’s health issues was a private, sensitive, and sometimes difficult conversation topic for a couple. For others, family planning decisions involved wanted as well as unwanted input from relatives, friends, religious leaders, and health workers. Low male acceptance of family planning had the potential to create adverse situations for some women, including personal safety risks, as shown in the following quote:

“There are some men whom after getting wind of their wives going for family planning, they start up fights and you will hear that a man has sent away the wife or beaten her up, when he finds out that she does family planning. So some men don’t perceive their wives in a good way to do family planning just as I mentioned to you some women go secretly and do family planning when the husband does not know.” --female participant

Some participants spoke of interlaced cultural beliefs and religious procreation expectations that encouraged married couples to have as many children as possible.

“People believe that if you have many children you may have wealth…. Some believe that if you have many sons, you have protection around you. At the same time the religion also part of it, affect the family planning. People believe that there are some few verses in the bible. People quote that God says that, “you go ye into the world and fill the world”. So, people just take it that way without knowing that if you have many children, the family planning actually is not you limit the number of children but it allows you to plan the family.” --male participant

Women, in particular, discussed male perceptions that contraceptive use provided an unfavorable opportunity for female sexual promiscuity, including involvement in prostitution.

“There are some who say family planning spoils women and that women go for family planning to be able to have extramarital affairs. Some men believe such women are promiscuous…. Some men believe family planning introduces women to promiscuity. They go for family planning so as to be free and not bear children.” --female participant

A few male participants also stated that the potential for family planning to require female nudity, in particular displaying of the female genitalia to someone other than a spouse, was problematic:

“…Some believe that it is only the husband who is supposed to see the nakedness of a woman, so some methods require that the privacy [sic] of a woman may be seen. So, some cultural beliefs do not accept those like the IUCDs [intrauterine contraceptive devices], mostly those ones.” --male participant

The most mentioned benefit of family planning was that it allowed the parents to care adequately for children they already had. Overall, the majority of participants expressed that the economic realities that came with providing food, clothing, shelter, and basic education to offspring largely influenced views and actions related to controlling family size and birth spacing. In some instances, family planning permitted participants’ own educational and occupational advancement. A less common perspective, mostly held by men, was that family planning improved a woman’s health. They indicated that by bearing fewer children, women were less likely to have to deal with depleted energy/ fatigue, added daily life stressors, and risk for vaginal infections or other reproductive health issues (e.g., cervical cancer).

Real or perceived level of partner support encouraged open or covert initiation as well as continuation of family planning by women. Decisions to disclose either intentions to use or actual use of family planning varied; however, some participants emphasized that ideally both parties needed to agree on family planning use. Descriptions of male involvement in family planning highlighted men’s reliance on women to educate and inform them on method options and possible health risks. Frequently, women as well as men incorporated stories based on others’ experiences heard about first hand or via hearsay to stress advantages or disadvantages of particular family planning methods. Descriptions of family planning risks centered primarily on side effects, contraceptive failure, birth defects, inability to conceive later, and increase in female sexual promiscuity. Overall, participants agreed that even where the decision to use family planning was arrived at jointly, women chose the actual method used in consultation with health workers.

In terms of their ring use experiences, women described a variety of ring disclosure approaches that they used with a sexual partner. Some discussed ring use with their partners before enrolling in the study. The partner already knew that the woman had been using family planning or he had shared with her that he was in favor of them doing so. Women in such scenarios spoke of the ability to openly (being free to) discuss ring use with their partners. Other women explained that concerns about something happening/going wrong motivated disclosure prior to or shortly after ring initiation. For a few women, in-depth interview selection prompted ring use disclosure. Both women and men emphasized the research context that introduced the contraceptive ring into the community as well as the possibility of unknown negative outcomes mentioned above. A few participants misstated that the ring’s efficacy had not yet been established; hence, the reason for undertaking the study. Lastly, a few women wanted to test (challenge) if their partners could feel the ring and withheld ring use disclosure for several months, while others had to disclose ring use because the partner felt the ring during sexual intercourse.

“I was afraid to tell him so I just came in the first month without telling him and he did not feel anything when we were having sex. I was afraid he would feel it but the doctor assured me that he would not feel it though I felt it was not true. So, I decided to give it a try by not telling him in the first and second month. In the second month, I decided to tell him that I was going to [name of local hospital omitted] and he asked me what I was going to do there then I told him that there was a research…a family planning method…was being inserted in the vagina, and I used it for a month but had not experienced any side effects. Then he asked me what I thought about it. I told him that I thought it would be a good thing if I would not have any infection and then he accepted. But I think if I told him in the beginning he would have declined.” --female participant

### Feeling “Normal”: Family planning needs to be compatible with the body

Participants emphasized the importance of a contraceptive method’s compatibility with the body. In discussing ring use experiences, most women mentioned placement issues during the first one to two months of ring use. In general, improper placement led to the ring shifting down or partially slipping out, physical discomfort (foreign body sensation), or mental distress that it would come out entirely. In such cases, assistance from study staff was sought and women said that they received additional instruction on how to insert the ring so that it was positioned correctly (deep enough). A few women talked about instances in which the ring completely slipped out (i.e., full expulsion). The strenuousness of activities at the time of expulsion ranged from carrying heavy objects, performing chores (as exemplified by the quote below), having sexual intercourse, to sleeping.

“What I do not like is that when I first inserted it, it kept on moving from its original place and it is like it is something that slowly moves from its original position until it eventually falls out when one does chores where you do not balance properly, where the thighs do not balance….It could come out in a place where you cannot easily pick it (laughs) maybe where there are people….”--female participant

Despite initial concerns, including beliefs that the ring would migrate (get lost inside, get pushed in, go all the way to the stomach), and that medicines inserted vaginally would cause infections (dirt) or bring about pain and discomfort, most reported ring use issues were temporary or minor, especially in comparison to those experienced with injectable and oral contraceptives.

Most participants indicated that they experienced little to no negative side effects during their ring use. In general, those who experienced side effects explained that problems that occurred with the onset of ring use either decreased or disappeared entirely after the second month. The most common side effects mentioned were nausea, dizziness, headaches, increased fatigue, lower back pain, and abdominal pain. Some women and men observed changes in a woman’s weight after initiating ring use. Most reacted positively toward these changes in weight, especially those who had experienced undesired weight gain with oral and injectable contraceptives. While weight loss was most common, weight gain also occurred for a few women.

Almost all participants held the perspective that unless a future ARV-based ring to prevent HIV infection also served a contraceptive purpose, uptake by women in their community would be low and acceptance by male partners would similarly be low. Overall, women viewed the ring as easy to use once they established comfort with vaginal insertion and removal, and achieved proficiency with ring placement, insertion and removal. In particular, those satisfied with the ring indicated that they liked the convenience of inserting and removing the ring themselves as opposed to visiting the clinic or hospital to have it done by a doctor. However, there was also displeasure about vaginal insertion and removal.

“What I don’t like about using it is removing it. When removing, it I see some dirt, which is not pleasing me. I don’t like [removing it] and also inserting it. When you want to insert it, you must look for a private place. You must be someone who has to do things in secrecy. Even when you are with people, you have to think of how you will insert it.” --female participant

For men, disliking the ability to feel the ring during sexual intercourse was an issue. Only one woman stated that she did not like having sex with the ring because it reduced her libido (cooled her off). Some of the men indicated that using the ring necessitated changes in sexual activity as shown by the following quotes:

“…It somehow caused some bruises. I do not know but I have just found out that …you know the normal way you play sex, there are some hindrances…. you know you tend to forget there is that thing inside and then you only realize the impact later. It increases infection somehow of course depending [on the way you play sex]. This one I would say to be honest, maybe we did not negotiate so much thinking of the ring…. Now when having sexual intercourse, a manner in which you need to play without actually rubbing the ring.” --male participant

Mention of the ring’s compatibility with the body was closely tied into women’s menstruation experiences. Both women and men explained that menstruation is natural and that suppression of women’s menstrual cycle is unhealthy (not normal). Most women indicated that the flow, volume, and duration of their menstrual cycles improved after switching to the ring. Women who were using an injectable contraceptive at enrollment were more likely to be pleased with “seeing” a normal menses return (i.e., a lighter or shorter flow, on regular, monthly basis). Some women indicated that during months 1 and 2 of ring use, they encountered increased or prolonged bleeding, or painful menstrual cycles before their periods normalized, while a few indicated that this was an ongoing issue. The latter type of users stated that this mainly contributed to their dislike of the ring. Awareness of menstrual issues by these women’s partners was also likely to lead to negative views toward the ring.

“…what I can say, my partner has always complained about pains in the back. That is what she has always complained of. I have always noticed long days of menstruation flow. Yes, there have been delays in the menstruation flows when they come my observation has always been that during that time she complains of headache and pains on the back, tiredness if it is the effect of the ring then that is where I can say the ring may be bad.” --male participant

### “How well does it really work”: Grappling with fertility control

Participants expressed discomfort with the uncertainties of fertility control as well as knowledge about how well the ring really worked. Women and men voiced concerns about the ring’s ability to prevent pregnancy, undisclosed risks for unplanned pregnancy while using the ring, plus the possibility for future fertility issues after stopping ring use. Some questioned how the design (i.e., an open ring) could work to prevent pregnancy. Others who thought that efficacy had not yet been established focused on the investigational research aspect of ring use. While not accurate, a few expressed relief that the ring was hormone-free. They believed that the ring offered local/topical prevention rather than a systemic one illustrated by the following quote:

“The ring is good…. [It] does not work in the blood like other methods of family planning. It just works around the part it has been placed. It directly works there. Mm, that in case you remove it then you will still be ok. That is what is good about it.” --female participant

One male participant revealed that some possible misunderstandings regarding duration of ring use as well as storage might have occurred.

“And how long this ring should take there [inside the vagina] … because sometimes I see it is being removed, put in the water, bring it here. Of course, I asked, and then I was told that it should be removed and you should bring it here.” --male participant

Three out of the four women diagnosed with a pregnancy described an insertion delay (one day to two weeks) after the 7-day ring free part of the cycle. The fourth woman indicated that she had removed the ring after the first 7 days of the 21-day use cycle to show it to her husband.

“I removed it and showed it to him and he told me to reinsert it but I did not do that. I told him that we were instructed to reinsert it after 21-days. My husband told me to return it…. He had read the consent form and understood and so when he told me to reinsert it, I lied to him that what we had been told was different from that and I lied that I had inserted it…. I don’t know if it was the devil, the devil tempts people at times. Initially, I felt bad since I got pregnant and my child is still very young, but after counseling, I realized it was God’s plan.” --female participant

## Discussion

Examination of the lived experience of contraceptive ring use may provide valuable insights on personal, social, cultural, situational, and other contextual factors influencing uptake and use of rings, particularly in settings where the technology is novel. Consideration of the meaning that participants themselves make of their ring use experiences within the social context of their everyday lives is also critical. Our qualitative study found that users’ experiences with the contraceptive ring reflected a broader family planning paradigm. In general, the essential structure of contraceptive ring experience for users centered around three themes: latitudes and drawbacks of family planning (being free); a family planning method needs to be compatible with a woman’s body (feeling normal); and dealing with fertility control uncertainties (how well does it really work).

Specific dimensions within two of these themes, being free and being normal may be less likely considered in developing biomedical technologies targeted at women for preventing pregnancy and ultimately for preventing HIV or other sexually transmitted infections with and without contraceptive co-formulation. The majority of the aspects of the third theme, how well does it really work, are central to clinical trials; however, they highlight the importance of the intersection between user certainty and knowledge of fertility control as well as complete information, clear use instructions, and practical use. Descriptions of ring use by three out of the four participants who became pregnant during their ring use period hints that mistakes in use or possibly unstated pregnancy intentions account for most of the pregnancies rather than unintended from method failure, which is consistent with findings from other studies [17].

Our findings suggest that ring uptake and use may be influenced by partner support, a user-friendly intuitive design, short- and long-term side effect concerns and efficacy implications. We note overlap between our findings and some of those shown for an analysis of Demographic and Health Surveys collected between 2005 and 2014 for 52 developing countries [18]. The reported top five reasons for not using a contraceptive method were side effects and health risks concerns, infrequent or no sex, opposition by others close to them, breastfeeding and/or menstruation not resumed after birth, and not being married (i.e., not willing to risk social disapproval potential associated with seeking family planning services) [18]. A study conducted in the city slums of Nairobi, Kisumu, and Mombasa found that poor family planning service utilization was due to women’s socioeconomic level and religious background, partner’s approval, knowledge about family planning services, and perceptions about the quality of family planning services, including staff friendliness [19].

Albeit a novel contraceptive technology, most women and men in our sample were satisfied with the ring. Both female and male participants held the perspective that a family planning method that “fit” a woman’s body was desirable. In comparing the ring with other contraceptive methods, women noted that side effects were minimal or manageable. Side effects centered predominantly on physical discomforts, such as headaches, fatigue, and dizziness although a few women reported menstruation problems.

While less explicit, this corporeal experience of “fit” extended to some men being able to feel the ring during sex. Males emphasized that they were in favor of ring use provided that they had little to no effect on a woman’s sexual desire/receptivity or her ability to conceive after terminating use. However, it is reasonable that men would discourage or not permit their partners to use a ring that interfered with male sexual performance and pleasure. Consistent with the literature, our findings also suggest that ring use (as with other forms of contraception) may afford women sexual freedoms that challenge gendered views regarding Kenyan women’s sexual morality and monogamy, as well as their sexual agency [20,21]. Nonetheless, there is a dearth of information in the literature regarding the male attitudes toward and experiences with rings.

Some participants expressed unease with vaginal insertion and removal of the ring. They cited underlying sexual mores discouraged women from touching their own genitalia and cultural beliefs regarding female nudity and spousal privilege. Tanner and colleagues [22] suggest that the social construction of women’s bodies as shared space requires that female-initiated (as opposed to female-controlled given gender power imbalances) microbicides need to consider the social context of settings where they will be used. Moreover, despite attempts to engage men in family planning, family planning clinics are still largely gendered spaces. Contraceptive-use paradigms may further place contraceptive responsibility predominately on women.

Our findings further suggest that improper fit and placement challenges may dissuade ring use. Participants indicated that ring slippage created both physical and mental discomfort. Partial and full expulsion occurred while sleeping as well as while engaging in tasks such as squatting, lifting heavy objects, or sexual activity, which further emphasizes possible fit-issues for some women. A one-size ring, assumes a uniform vaginal cavity size. In a United States-based study, concerns about the ring getting lost inside or falling out reduced willingness to try the ring [23]. Before initiating ring use, informing users that practice may be required and that finding a comfortable fit may not be associated with insertion and removal are important. Clinical staff may assist with user proficiency provided, of course, those users who are experiencing problems seek such assistance.

While some of the women and men in our study told us that they felt the ring during sexual intercourse, they also reported observing little to no negative influence on a woman’s libido or a male’s performance. One study found that discontinuation of ring use is higher if women feel the ring inside the vagina, they experience sexual interference, or expulsion occurs [24]. Women in our study seemed to appreciate the self-insertion/removal aspect of the ring. Although they had to return to the clinic every month to pick up a new ring and complete an adherence visit, the convenience of not having to rely on a medical provider to insert it made the ring an attractive contraceptive method.

Ring acceptability studies have centered on examining preferences related to its physical properties (size, thickness, color, odor, etc.), assessing willingness to use, and measuring adherence, tolerability and satisfaction constructs. After undertaking this study, it became clearer that assessment of acceptability might benefit from a multi-pronged approach. Notional acceptability is useful for getting an idea about initial receptivity toward a new product, including one licensed but novel to a specific setting, or one in development based only on information received about it. While giving potential users the opportunity to visually and manually inspect a prototype ring is optimal in this case, interpretation of acceptability warrants caution. In a study of contraceptive ring and vaginal lubricant users, the investigators found that focus group participants relied on their prior product experiences and sensory perceptions to inform their perceptions regarding product properties and efficacy [19]. This process, known as framing, may make it difficult for potential users to provide meaningful input on future ARV-based rings, especially if such rings differ greatly from existing expert user-knowledge; hence, the importance of knowing the contraceptive-use paradigm before introducing a new product.

Acceptability based on actual experience with a new product or technology (i.e., acceptability in *praxis*) in clinical trials or product testing shifts notional acceptability into a conditional use context. Such conditions, which typically are established in the clinical trial or research design, involve some temporary duration of use that is influenced by multiple factors at the participant level (e.g., product satisfaction, tolerability, partner support, efficacy, cost, reproductive intentions/desires, and particularly in disadvantaged settings, how incentivized). Acceptance/adoption (the believer), which may not be feasible to assess in phase II and phase III clinical trials, refers to invested use that is based on conscious choice and practice. With product acceptance, continued use is situational and in addition to the factors identified for acceptability in *praxis*, issues of access are critical. Strategies for assessing and optimizing adherence, however, are still required.

Ring use requires more than just a woman familiarizing herself with insertion and removal procedures or the use schedule. The corporeal experiences of women and men in our study suggest that the learning phase may be associated with both ring placement and sexually activity. In addition, the mental task of remembering on-ring and off-ring cycles may not be as easily accomplished; yet, ongoing or long-term suppression of menstruation may not be desirable for all women. In comparing the ring to injectable and oral contraceptives, several of our participants mentioned the noticeable improvement in a woman’s libido. This appears to be an understudied aspect in ring microbicide product development. As Higgins and Smith [9] point out, consideration should be given to how methods may influence the user’s sexual experiences, especially given that this experience may then have broader consequences for family planning preferences and practices. Additionally, adherence may be improved if extra efforts are taken to ensure that women as well as their partners fully comprehend drugs that a ring contains and why correct use is important. Assessing how instructional exceptions (e.g., ring removal during sex) may affect comprehension may be required.

Based on our findings, partner and community approval needs to be further explored given their probable role in ring uptake. An analysis of contextual influences on modern contraception in six African countries showed that level of community approval of family planning had a larger effect on contraceptive use than did the perceived approval of the woman’s partner [25]. While not mentioned by our participants, examination of the political context of reproduction (more children increase voting block) in this setting may be required. Lastly, as has been proposed by others, health researchers and professionals also need to be more aware of their own contexts and that they work towards carefully identifying at least one user-specific context layer that can be used to create a multilayer approach [26].

### Limitations

Despite the potential usability of our findings, our study has several important limitations. It is unknown if a larger sample size may have strengthened the “typicality” of our findings or expanded the themes identified. We acknowledge oversight of not interviewing women who decided to stop using the ring or who discontinued ring use for medical reason other than pregnancy. Additionally, women in our study may have been more inclined to communicate ring use with their partners despite differences in disclosure timing and motivations. Likewise, the men we interviewed may have been more supportive of family planning than may be characteristic of other men in the community. Optimally, future research should consider inclusion of these categories of participants. A team-based qualitative research offers both advantages and disadvantages. Varying interviewer skills could affect interview quality, including the level of detail and clarity in information obtained. Matching participants and interviews by gender was not always possible. Nonetheless, such interviewing situations were important in gaining insights regarding the various ways in which cross-gender talk occurs. Men, interviewed by a female interviewer, may have recalled and recounted experiences that differed from those disclosed with a male interviewer.

## Conclusions

Awareness of the multiple and often overlapping contexts (personal, social, cultural, situational, corporeal, etc.) in ring user experience may help inform specific ring development, education, and promotion approaches. Educating researchers and participants on aspects of ring use that may be absent or have limited coverage in product informational brochures or product counseling materials may be critical.

## Figures and Tables

**Table 1:** Characteristics of ring study in-depth interview participants, Kisumu, Kenya, 2014–2015.

Demographic variable	Total (n=44)	Women (n=24)	Men (n=20)
	n (%)	n (%)	n (%)
Age groups			
18–20	7 (15.9)	6 (25.0)	1 (5.0)
21–24	9 (20.5)	6 (25.0)	3 (15.0)
25–28	14 (31.8)	8 (33.3)	6 (30.0)
≥ 29	14 (31.8)	4 (16.7)	10 (50.0)
Mean age (range)	28 (18–58 years)	25 (18–24 years)	33 (18–58 years)
Ethnic/tribal group			
Luo	35 (79.5)	18 (75.0)	17 (85.0)
Non-Luo	9 (20.5)	6 (25.0)	3 (15.0)
Marital status			
Not married*	6 (13.6)	5 (20.8))	1 (5.0)
Married/living as married	38 (86.4)	19 (79.2)	19 (95.0)
Religion			
Roman Catholic	7 (15.9)	6 (25.0)	1 (5.0)
Other Christian	28 (63.6)	14 (58.3)	14 (70.0)
Other non-Christian	9 (20.5)	4 (76.7)	5 (25.0)
Highest education completed			
Primary or less	19 (43.2)	14 (58.3)	5 (25.0)
Secondary or higher	24 (54.5)	9 (37.5)	15 (75.0)

* Single, separated, divorced, and widowed
